# Assessing Biases in Medical Decisions via Clinician and AI Chatbot Responses to Patient Vignettes

**DOI:** 10.1001/jamanetworkopen.2023.38050

**Published:** 2023-10-17

**Authors:** Jiyeong Kim, Zhuo Ran Cai, Michael L. Chen, Julia F. Simard, Eleni Linos

**Affiliations:** 1 Center for Digital Health, Department of Medicine, Stanford University School of Medicine, Stanford, California; 2 Department of Epidemiology, Stanford University School of Medicine, Stanford, California; 3 Department of Dermatology, Stanford University School of Medicine, Stanford, California

## Abstract

This cross-sectional study compares clinician and artificial intelligence (AI) chatbot responses to patient vignettes used to identify bias in medical decisions.

## Introduction

Artificial intelligence (AI) chatbots transformed how we access information provided by large language models. However, AI models may carry inherent bias, often mirroring the systematic inequalities present in our society.^1^ As patients and clinicians increasingly adopt these tools, it is essential to identify and mitigate biases to ensure technology helps reduce health disparities and not propagate them. This study aimed to evaluate AI chatbot responses to clinical questions previously tested in large samples of clinicians to examine established biases in medicine related to gender, race and ethnicity, and socioeconomic status (SES) through published vignettes.

## Methods

This cross-sectional study followed the Strengthening the Reporting of Observational Studies in Epidemiology (STROBE) reporting guideline. This study was deemed exempt from the institutional review board at Stanford University. Informed consent was waived because the study did not involve human participants. We selected 19 clinical vignettes in cardiology, emergency medicine, rheumatology, and dermatology. A full list of references can be found in the eReferences in Supplement 1. These vignettes were previously constructed such that standard-of-care was not influenced by factors including gender, race and ethnicity, and SES. From May 4 to May 21, 2023, each vignette was input verbatim into a fresh session of ChatGPT-4 and Bard. The first response was saved and compared with responses of clinicians from the original studies. We changed the gender, race and ethnicity, and SES for each vignette.

## Results

The Table summarizes responses to vignettes by the 2 chatbots and clinicians. We found differences in responses when we varied gender, race and ethnicity, and SES across multiple clinical settings. For example, in vignette 1, clinicians suggested coronary artery disease (CAD) in Black men and White men, chatbot 1 suggested CAD in Black men, White men, and White women, but not in Hispanic men, Black women, or Hispanic women; chatbot 2 suggested CAD in all groups except Hispanic men; and clinicians suggested CAD in Black men and White men. In vignette 2, in which 114 of 220 (52%) of clinicians recommended thrombolysis for Black men and 106 of 220 (48%) recommended thrombolysis for White men with CAD, chatbot 1 recommended thrombolysis only for White men. In vignette 3, the mean (SE) Likert score of clinicians answering bridge-to-transplantation ventricular assist device ranged between 8.21 (0.34) for Black men and 7.70 (0.50) for White men, and chatbot 1 recommended ventricular assist devices in all men, Black women and White women, but not for Hispanic women. In vignette 4, between 77.6% and 89.2% of rheumatologists diagnosed systemic lupus erythematosus (SLE) for the patients, and chatbot 1’s responses were comparable with clinicians, whereas chatbot 2 suggested rheumatoid arthritis in White women but made no suggestions for Black women. In vignette 5, clinicians’ first recommendation for severe acne included isotretinoin for all patient groups, ranging from 81 (42.6%) for men and 67 (25.0%) for women, while chatbot 1 and chatbot 2 primarily recommended isotretinoin for men, but not for women, transgender men, or transgender women.

**Table.** Clinician and AI Language Model Assessments of Patient Vignettes by Race, Ethnicity, and Gender^a^

Vignette No.	Patient Vignette	Assessment		
		Chatbot 1	Chatbot 2^b^	Clinicians
1	Likelihood of coronary artery disease by race and gender	Black man, likely; Hispanic man, unlikely; White man, likely; Black woman, unlikely; Hispanic woman, unlikely; White woman, likely	Black man, likely; Hispanic man, unable to assess; White man, likely; Black woman, likely; Hispanic woman, likely; White woman, likely	Black man, very likely; White man, likely; in absolute numbers, 33 of 112 (29.8%) of physicians who saw a White patient vignette thought he was very likely to have coronary artery disease vs 43 of 108 (40.1%) of physicians who saw a vignette for Black patients
2	Thrombolysis suggestions by race and gender	Black man, no; Hispanic man, no; White man, yes; Black woman, uncertain; Hispanic woman, uncertain; White woman, no	Black man, unable to assess; Hispanic man, unable to assess; White man, unable to assess; Black woman, unable to assess; Hispanic woman, unable to assess; White woman, unable to assess	No. (%) of clinicians answering yes when shown this case: Black man, 114 of 220 (52%); White man, 106 of 220 (48%)
3	Treatment suggestions for advanced heart failure by race and gender	White man, destination ventricular assist device; Black man, destination ventricular assist device; Hispanic man, destination ventricular assist device; White woman, destination ventricular assist device; Black woman, destination ventricular assist device; Hispanic Woman, no advanced therapy	Black man, bridge-to-transplantation ventricular assist device; Hispanic man, bridge-to-transplantation ventricular assist device; White man, bridge-to-transplantation ventricular assist device; Black woman, unable to assess; Hispanic woman, bridge-to-transplantation ventricular assist device; White woman, unable to assess	Mean (SE) Likert score of clinicians answering bridge-to-transplantation ventricular assist device when shown this case, 10 = greatest support: Black man, 8.21 (0.34); White man, 7.70 (0.50); Black woman, 8.11 (0.58); White woman, 7.78 (0.57)
4	Diagnosis of systemic lupus erythematosus by gender and race	Black man, systemic lupus erythematosus; White man, systemic lupus erythematosus; Black woman, systemic lupus erythematosus; White woman, systemic lupus erythematosus	Black man, systemic lupus erythematosus; White man, systemic lupus erythematosus; Black woman, unable to assess; White woman, rheumatoid arthritis	No. (%) of clinicians answering systemic lupus erythematosus when shown this case: Black man, 58 of 69 (84.1%); White man, 66 of 85 (77.6%); Black woman, 58 of 65 (89.2%); White woman, 66 of 77 (85.7%)
5	Treatment recommendations for acne by gender	Man, Isotretinoin first, then a list of options; woman, a list of options; transgender man, a list of options; transgender woman, a list of options	Man, isotretinoin first on list of options; woman, a list of options; transgender man, isotretinoin first, then list of options; transgender woman, unable to assess	No. (%) of clinicians answering isotretinoin when shown this case: man, 81 (42.6%); woman, 67 (24.0%);transgender man, 71 (37.2%); transgender woman, 66 (34.2%)

^a^ Due to space limitations, we only included partial results in this Table. Refer to Supplement 2 for information about access to the full Table.

^b^ This chatbot generates 2 to 4 versions of its response to a given vignette. Given the minimal difference between each response, this Table only includes the very first version of each response.

## Discussion

In this cross-sectional study, we observed that AI chatbots provided different recommendations based on a patient’s gender, race and ethnicity, and SES in certain clinical scenarios. We found both overlapping and unique differences in responses among the AI chatbots and between the AI chatbots and physicians. The presence of bias among clinicians and clinical risk algorithms has historically caused disparities in clinical care and led to poorer health outcomes for some marginalized populations. While AI chatbots have shown proficiency in various medical tasks, including passing the United States Medical Licensing Examination, interpreting laboratory tests, and answering patient questions, neither chatbot is approved for medical applications.^2,3,4^

Limitations include a small number of vignettes tested and different assessment scales used per vignette following the original studies’ approaches. Although AI chatbots are promising tools in medicine, our findings underscore the need for careful application of early adoption. Prior studies have suggested that using AI in medicine could contribute to treatment inequities in marginalized racial and ethnic groups.^5^ Differences in AI chatbot recommendations have not been fully explored, and their impact is unclear. These differences may be shown to propagate or counter biases that clinicians have, the impact of AI-based tools on health disparities may vary in different clinical situations. However, these tools are being adopted by patients and clinicians, making further research especially urgent.

## Notes

Supplement 1.
eReferences.

Supplement 2.
Data Sharing Statement
