# Supplementary material for: Assessing Biases in Medical Decisions via Clinician and AI Chatbot Responses to Patient Vignettes
Source: JAMA Netw Open. 2023 Oct 17;6(10):e2338050. doi: 10.1001/jamanetworkopen.2023.38050 (PMC10582782; doi:10.1001/jamanetworkopen.2023.38050)
Supplement: Supplement 1. — eReferences. [file jamanetwopen-e2338050-s001.pdf]

Supplemental Online Content

Kim J, Cai ZR, Chen M, Fridman Simard J, Linos E. Assessing biases in medical decisions via clinician and ai chatbot responses to patient vignettes. *JAMA Netw Open*. 2023;6(10):e2338050. doi:10.1001/jamanetworkopen.2023.38050

eReferences

This supplemental material has been provided by the authors to give readers additional information about their work.

© 2023 Kim J et al. *JAMA Network Open.*

eReferences

The 8 studies that the vignettes were pulled are all cited in this supplemental material.

1. Green AR, Carney DR, Pallin DJ, et al. Implicit Bias among Physicians and its Prediction of Thrombolysis Decisions for Black and White Patients. *J Gen Intern Med*. 2007;22(9):1231. doi:10.1007/S11606-007-0258-5
2. Breathett K, Yee E, Pool N, et al. Association of Gender and Race With Allocation of Advanced Heart Failure Therapies + Editorial + Supplemental content. *JAMA Netw Open*. 2020;3(7):2011044. doi:10.1001/jamanetworkopen.2020.11044
3. Daugherty SL, Blair I V., Havranek EP, et al. Implicit gender bias and the use of cardiovascular tests among cardiologists. *J Am Heart Assoc*. 2017;6(12). doi:10.1161/JAHA.117.006872
4. Lutfey KE, Link CL, Marceau LD, et al. Diagnostic certainty as a source of medical practice variation in coronary heart disease: Results from a cross-national experiment of clinical decision making. *Med Decis Making*. 2009;29(5):606. doi:10.1177/0272989X09331811
5. Centola D, Guilbeault D, Sarkar U, Khoong E, Zhang J. The reduction of race and gender bias in clinical treatment recommendations using clinician peer networks in an experimental setting. *Nat Commun 2021 121*. 2021;12(1):1-10. doi:10.1038/s41467-021-26905-5
6. Haider AH, Sexton J, Sriram N, et al. Association of unconscious race and social class bias with vignette-based clinical assessments by medical students. *JAMA*. 2011;306(9):942-951. doi:10.1001/JAMA.2011.1248
7. Simard JF, Chaichian Y, Rizk N, Rector A, Feldman CH, Falasinnu TO. ARE WE MISSING LUPUS IN MALES? EVIDENCE OF COGNITIVE BIAS FROM A RANDOMIZED EXPERIMENT IN THE UNITED STATES. *Am J Epidemiol*. 2022;191(1):230. doi:10.1093/AJE/KWAB199
8. Zadu A, van Egmond S, de Vere Hunt I, Petion E, Cai Z, Yeung H, Simard J, Linos E. Prescribing Patterns for Acne in Transgender Compared to Cisgender Patients. Poster presented at American Academy of Dermatology 2023 Annual Meeting. March 17th, 2023; New Orleans, LA, USA.

© 2023 Kim J et al. *JAMA Network Open.*
