# Supplementary material for: Assessing Biases in Medical Decisions via Clinician and AI Chatbot Responses to Patient Vignettes
Source: JAMA Netw Open. 2023 Oct 17;6(10):e2338050. doi: 10.1001/jamanetworkopen.2023.38050 (PMC10582782; doi:10.1001/jamanetworkopen.2023.38050)
Supplement: Supplement 2. — Data Sharing Statement [file jamanetwopen-e2338050-s002.pdf]

Data Sharing Statement

Kim. Assessing Biases in Medical Decisions via Clinician and AI Chatbot Responses to Patient Vignettes. *JAMA Netw Open*. Published October 17, 2023.
doi:10.1001/jamanetworkopen.2023.38050

Data
Data available: Yes
Data types: Other (please specify)
Additional Information: will be in supplement. The authors are willing to share the full result table, which presents 19 vignettes, questions, and answers of the AI chatbots and clinicians from the 8 original studies, with those who make a reasonable request.
How to access data: will be in supplement
When available: With publication

Supporting Documents
Document types: None

Additional Information
Who can access the data: HTML files from chatgpt will be shared as supplement
Types of analyses: HTML files from chatgpt will be shared as supplement
Mechanisms of data availability: HTML files from chatgpt will be shared as supplement
Any additional restrictions: none
